# Three-dimensional printing-assisted interventional therapy for thoracogastric right main bronchial fistula

**DOI:** 10.1055/a-2274-5928

**Published:** 2024-03-14

**Authors:** Zhen Li, Yifan Li, Zongming Li, Huibin Lu, Xin Li, Xinwei Han, Kewei Ren

**Affiliations:** 1 191599 Department of Interventional Radiology, The First Affiliated Hospital of Zhengzhou University, Zhengzhou, China

Based on 3D printed models, the size, location, and morphology of thoracogastric airway fistulas can be more accurately assessed, which can help physicians better determine the size and location of stents or occluders 1 .

A 48-year-old man with a thoracogastric right main bronchus fistula after radical resection of esophageal carcinoma underwent esophageal stent placement. However, the stent needed to be removed due to stent migration. Considering the insufficient conformity of the stent on the digestive side of the patient and the large number of durable complications associated with tracheal stent implantation 2 3 , it was decided to seal the fistula with an occluder 4 . The right main bronchus and residual stomach were abnormally communicative after the stent removal ( Fig. 1 ). To assist with the selection of the occluder model and guide the operation, we constructed an in vitro 3D model of the patient's thoracogastric airway fistula ( Fig. 2 , Video 1 ). Subsequent reexamination confirmed that the occluder had been positioned satisfactorily ( Fig. 3 ).

**Fig. 1:**
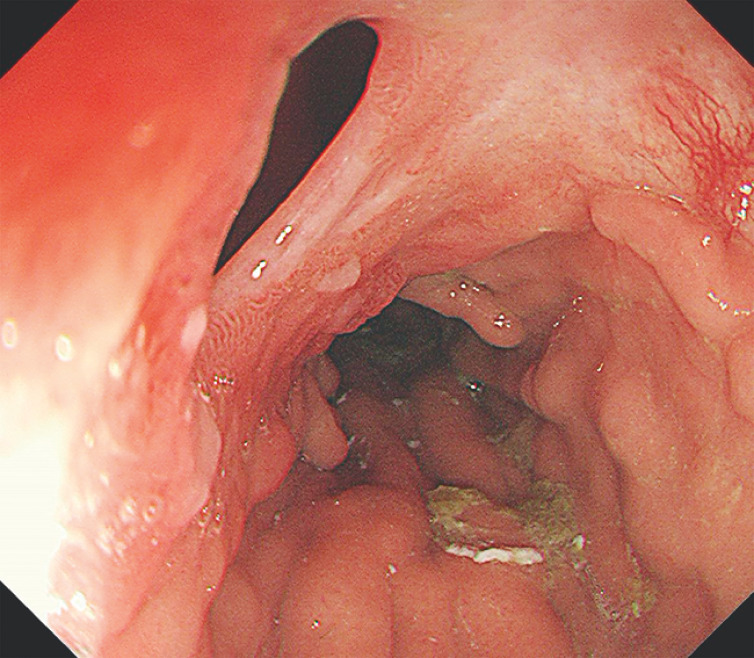
Gastroscopy showed residual gastrosomatic fistula.

**Fig. 2:**
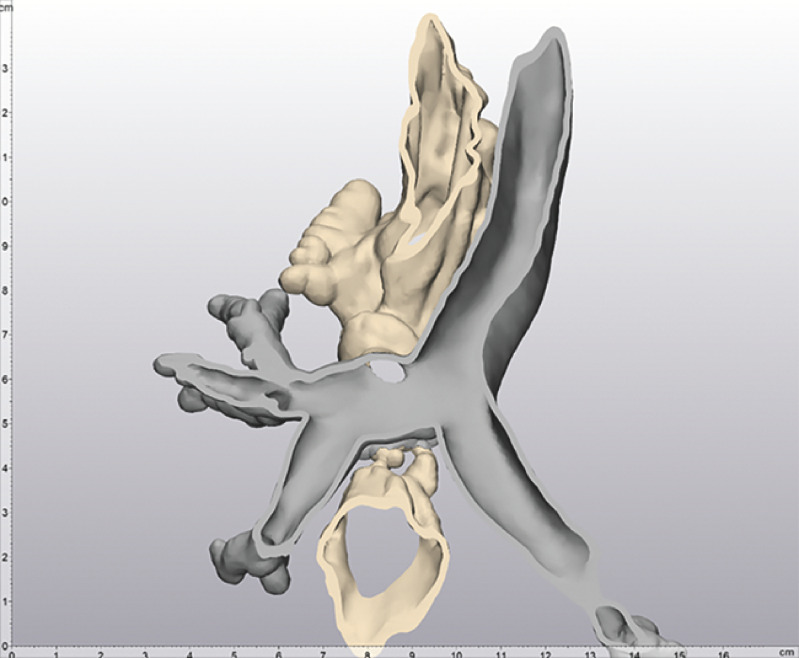
To assist with the selection of the occluder model and guide the operation, we constructed an in vitro 3D model of the patientʼs thoracogastric airway fistula.

**Fig. 3:**
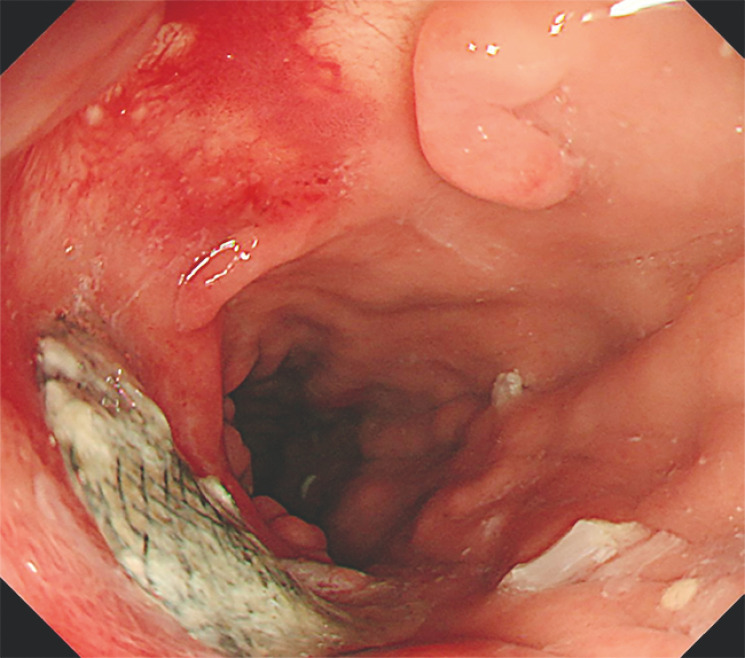
Subsequent reexamination confirmed that the occluder had been positioned satisfactorily.

3D printing-assisted occluder and double Y-stent successively occluded right thoracogastric main bronchial fistula.Video 1

The patient presented with symptoms of fever, cough, and sputum recurrence 10 months post-surgery, leading to the suspicion that the occluder had become dislodged. A digital radiography examination revealed that the occluder had fallen off into the colon and was discharged through the anus ( Fig. 4 ). Bronchoscopy found that a fistula with a diameter of about 2 cm was visible at the proximal right bronchus inferior carina. 3D printing was performed again for fistula condition. The stent type was selected based on the 3D printed model and simulated in vitro occlusion. A double Y-stent was placed to avoid the risk of occluder migration ( Fig. 5 ). Up to 9 months after the procedure ( Video 1 ), the patient had no related symptoms. The imaging results indicated that the patientʼs stent fit was satisfactory. Based on 3D printing, more accurate treatment options are provided for in vitro evaluation of fistula size and location and selection of occluders and stents with appropriate diameters.

**Fig. 4:**
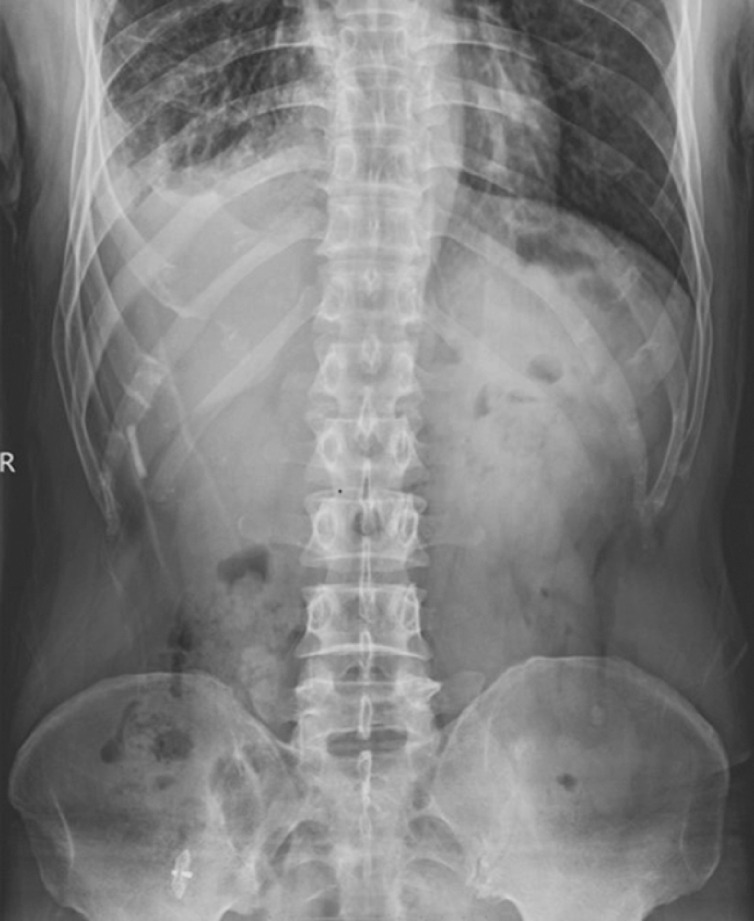
Digital radiography examination revealed that the occluder had fallen off into the colon.

**Fig. 5:**
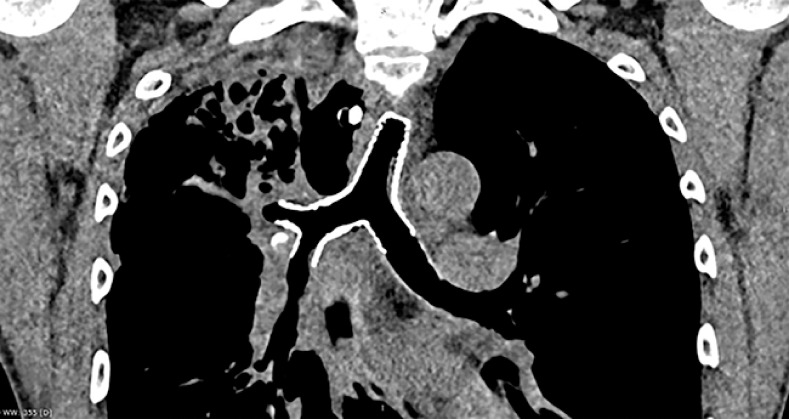
Double Y-shaped stent implantation was employed.

Endoscopy_UCTN_Code_TTT_1AO_2AI

## Notes

Endoscopy E-Videos https://eref.thieme.de/e-videos
*E-Videos* is an open access online section of the journal *Endoscopy* , reporting on interesting cases and new techniques in gastroenterological endoscopy. All papers include a high-quality video and are published with a Creative Commons CC-BY license. Endoscopy E-Videos qualify for HINARI discounts and waivers and eligibility is automatically checked during the submission process. We grant 100% waivers to articles whose corresponding authors are based in Group A countries and 50% waivers to those who are based in Group B countries as classified by Research4Life (see: https://www.research4life.org/access/eligibility/ ). This section has its own submission website at https://mc.manuscriptcentral.com/e-videos .
